# Editorial: World Chagas disease day 2022

**DOI:** 10.3389/fcimb.2023.1209531

**Published:** 2023-05-01

**Authors:** Giuseppe Palmisano, Nobuko Yoshida

**Affiliations:** ^1^ Instituto de Ciências Biomédicas, Universidade de São Paulo, São Paulo, Brazil; ^2^ Escola Paulista de Medicina, Universidade Federal de São Paulo, São Paulo, Brazil

**Keywords:** *Trypanosoma cruzi*, Chagas disease, pathogenesis, treatment, vaccine, resistance to complement

*Chagas disease is caused by the protozoan parasite* Trypanosoma cruzi. Most of the estimated 7 million people infected with *T. cruzi* live in Latin America, but the increasing number of infected individuals in many developed countries where the disease is not endemic, due to migration, is a matter of concern. Treatment of *T. cruzi* infection is restricted to two drugs, with limited efficacy in chronic Chagas disease and considerable side effects. A vaccine is currently unavailable and several questions related to pathogenesis and disease progression are not fully understood. We have received seven contributions for this Research Topic entitled *World Chagas Disease Day 2022*.

Aiming at collecting first data on the potential risk of transfusion-associated transmission of *T. cruzi* in Germany, Ullrich et al. analyzed 305 blood donors originating from Chagas disease endemic Latin American countries. All samples tested seronegative for *T. cuzi*-specific antibodies. Another study to gain knowledge about potential risk factors for *T. cruzi* infection in Germany was performed by Wirth et al. They conducted a cross-sectional, questionnaire-based study in six cities in Germany, between March 2014 and October 2019, with participation of 168 Latin American migrants. Most participants were also willing to donate blood and organs and a quarter of them had previously donated blood. One out of 56 serologic tests performed was positive. The seropositive female participant had a negative PCR test and no signs of cardiac or other organ involvement. Both studies point to the relevance of risk-adapted serology-based blood donor screenings in Germany, to prevent a risk of occasional transmission of *T. cruzi*.

Searches for an alternative drug to treat *T. cruzi* infection have been pursued by many research groups. Based on the fact that *T. cruzi* is unable to synthesize purines *de novo*, relying on a purine salvage pathway, Barnadas-Carceller et al. evaluated the anti-*T. cruzi* activity of 23 purine analogs with different substitutions in the complementary chains of their purine rings. By screening their capacity to inhibit parasite growth, their toxicity in Vero and HepG2 cells, and their inhibitory effect on amastigote development, eight compounds were found to have specific anti-*T. cruzi* activity. The results showing that specific substitutions, like the presence of benzene groups in the C8 position of the purine ring, are associated with high and specific anti-parasitic activity, point to the potential of synthetic nucleoside analogs for alternative treatment of *T. cruzi* infection.

Concerning *T. cruzi*-induced cardiac pathogenesis, which may be associated with alteration of host gene expression, Rayford et al. evaluated dysregulation of a class of sncRNAs called piRNAs during early phase of *T. cruzi* infection in primary human cardiac fibroblasts by RNA-Seq. During parasite challenge, 441 unique piRNAs were differentially expressed and of these 29 were known. In silico analysis showed that several of these piRNAs were computationally predicted to target and potentially regulate the expression of genes including *SMAD2, EGR1, ICAM1, CX3CL1*, and *CXCR2*, which have been implicated in parasite infection, pathogenesis, and various cardiomyopathies. It is suggested that piRNAs may play important roles in pathogenesis and can serve as potential biomarkers and therapeutic targets.

Attempts to develop a vaccine against *T. cruzi* have been made over the years. To date, however, no vaccine is available, either prophylactic or therapeutic. Jones et al. tested a vaccine-linked chemotherapy strategy in a mouse model of chronic *T. cruzi* infection to evaluate the effect on cardiac function. BALB/c mice were infected with *T. cruzi* and were treated beginning 70 days after infection with a low dose of benznidazole (BNZ) and either low or high dose of vaccine, consisting of recombinant Tc24-C4 protein and a TLR-4 agonist adjuvant. Cardiac health of treated mice, of control untreated mice, or mice that received only one treatment, was monitored throughout the course of treatment by echocardiography and electrocardiograms. Endpoint histopathology was performed approximately 8 months after infection to measure cardiac fibrosis and cellular infiltration. Vaccine-linked chemotherapy improved cardiac function and induced durable antigen-specific immune responses.

Identification of biomarkers for Chagas disease progression is a matter of considerable interest. A study by Soprano et al. indicates that antibodies specific for sulfated epitopes (sulfotopes) of *T. cruzi* glycoconjugates might be considered biomarkers of Chagas disease progression. The authors refer to the major *T. cruzi* antigen cruzipain (CZ), humoral immune response to sulfated-Cz elicited in *T. cruzi* chronically infected subjects and the involvement of Cz-sulfotopes-specific antibodies in the immunopathogenesis in an animal model. Reference is also made to higher levels of IgG2 antibodies specific for sulfated glycoproteins and sulfatides in sera from chronically infected individuals with mild disease, as compared to those with more severe forms of the disease.

The infective forms of *T. cruzi* are resistant to destruction by the complement system of the mammalian host, and this enables the parasites to survive and go through cycles of cell invasion, intracellular replication and evasion. Rossi et al. selected a population of *T. cruzi* epimastigotes through two rounds of exposure to normal human serum, to reach 30% survival (2R population), and compared the characteristics of these parasites with those of non selected wild type (WT) population. The rate of differentiation to infective metacyclic trypomastigotes was higher in 2R parasites than in WT population. Resistance to complement-mediated lysis of 2R metacyclic trypomastigotes increased by two-fold and the parasites were at least three times more infective to mammalian cells. Extracellular vesicles from 2R parasites could transfer the invasive phenotype to the WT population. The higher infectivity of 2R parasites was preserved in trypomastigotes derived from cultured cells, and the rate of parasite release from infected cells was higher as compared to WT population.

Overall, this Research Topic brings articles focused on relevant aspects of *T. cruzi* infection, which are of particular interest to the Chagas disease research community.

## Author contributions

All authors listed have made a substantial, direct, and intellectual contribution to the work and approved it for publication.

